# Planetary Health Diet Index and self-reported kidney stones in US adults: mediating role of high-density lipoprotein cholesterol

**DOI:** 10.3389/fnut.2025.1609626

**Published:** 2025-11-10

**Authors:** Qinglong Yang, Haolin Chen, Nan Luo, Hanyuan Lin, Haoxian Tang, Jingtao Huang, Xuan Zhang, Wenqiang Liao, Gaoming Hou, Yuxue Lin, Zexuan Liu, Xuxia Sui, Qingtao Yang

**Affiliations:** 1 Department of Urology, The Second Affiliated Hospital of Shantou University Medical College, Shantou, Guangdong, China; 2 Department of Thyroid, Breast and Hernia Surgery, General Surgery, The Second Affiliated Hospital of Shantou University Medical College, Shantou, Guangdong, China; 3 Shantou University Medical College, Shantou, Guangdong, China; 4 Department of Psychiatry, Shantou University Mental Health Center, Shantou, Guangdong, China; 5 Department of Cardiology, The First Affiliated Hospital of Shantou University Medical College, Shantou, Guangdong, China; 6 Department of Sports Medicine and Rehabilitation, Peking University Shenzhen Hospital, Shenzhen, Guangdong, China; 7 Department of Bone & Joint Surgery, Peking University Shenzhen Hospital, Shenzhen, Guangdong, China; 8 Department of Pathogenic Biology, Shantou University Medical College, Shantou, Guangdong, China

**Keywords:** Planetary Health Diet Index, kidney stones, high-density lipoprotein cholesterol, NHANES, association

## Abstract

**Background:**

Kidney stone is a universal health concern, with its incidence influenced by dietary habits. The Planetary Health Diet (PHD) benefits human health and the environment.

**Objective:**

This study aims to examine the relationship between the Planetary Health Diet Index (PHDI) and self-reported kidney stones and to explore the mediating role of high-density lipoprotein cholesterol (HDL-C).

**Methods:**

This study included 19,249 participants (≥20 years old) from the National Health and Nutrition Examination Survey (NHANES) from 2007 to 2018. Kidney stone diagnoses were self-reported. Higher PHDI scores represented greater compliance with the PHD. The statistical analyses encompassed a weighted multivariable logistic regression model, restricted cubic spline curve analysis, mediation analyses, subgroup analysis, and sensitivity analysis.

**Results:**

In the fully adjusted model, each 10-point rise in PHDI was tied to an 8% decrease in self-reported kidney stone risk (OR, 0.92; 95% CI, 0.87–0.97). Participants in the top quintile of PHDI had a 25% lower risk of self-reported kidney stones than those in the bottom quintile (OR, 0.75; 95% CI, 0.58–0.96). HDL-C mediated 6.0% of the correlation between PHDI and self-reported kidney stones.

**Conclusion:**

Planetary Health Diet Index reduced the risk of self-reported kidney stones, with HDL-C partially mediating this effect.

## 1 Introduction

Kidney stone represents a prevalent urological disorder, manifesting in roughly 1 in 11 persons in the US (1), with an extremely high recurrence rate of around 50% within a decade (2). It has been observed to contribute to the development of chronic kidney disease (3), urinary tract infection (4), end-stage renal disease (5), cardiovascular disease (CVD) (6), and osteoporosis (7), posing remarkable health challenges. In addition, the diagnosis and treatment place a substantial economic strain on healthcare systems (8). Therefore, developing effective prevention strategies for kidney stones is of paramount importance.

The food system’s influence on the environment and human health is profound and complex. While feeding a growing global population, the current system also leads to environmental degradation and public health risks. To address this, the EAT-Lancet Commission published a proposal in 2019, namely the Planetary Health Diet (PHD), which promotes plant-based foods and recommends the moderation of animal-based products (9). In addition, the Planetary Health Diet Index (PHDI) is a method to quantify an individual’s or population’s adherence to the PHD guidelines (10–12). Notably, the PHD differs from alternative dietary patterns in that it promotes planetary sustainability by lowering greenhouse gas emissions, freshwater consumption, and the usage of nitrogen and phosphorus (11, 13), making it increasingly relevant amid growing environmental concerns.

Additionally, the PHD was linked to a decreased risk of several illnesses, including CVD (14), asthma (15), metabolic dysfunction-associated fatty liver disease (16), and diabetes mellitus (DM) (17). However, its association with kidney stones remains unclear. Prior studies have demonstrated the repercussions of diet on the formation of kidney stones, with interventions such as reducing processed and red meat intake (18) and increasing fruit and fiber consumption (19) effectively lowering risk. Given that the PHD emphasizes plant-based foods and moderate animal product intake, adherence to the PHD may reduce kidney stone risk. This would provide a scientific basis for dietary interventions that both prevent kidney stones and promote environmental sustainability, which is of substantial importance.

High-density lipoprotein cholesterol (HDL-C), colloquially termed “good cholesterol,” facilitates the transport of excess cholesterol from peripheral tissues to the liver (20). As demonstrated by earlier research, PHDI and HDL-C correlated positively (21). Moreover, elevated HDL-C was thought to reduce kidney stone risk (22). Thus, we hypothesize that HDL-C may mediate the linkage between PHDI and the development of kidney stones.

Our study utilized the National Health and Nutrition Examination Survey (NHANES) database to probe the nexus between PHDI and self-reported kidney stones in US adults, with specific attention directed toward the hypothetical mediating function of HDL-C.

## 2 Method

### 2.1 Data sources

The National Center for Health Statistics (NCHS) spearheads the NHANES, which is a sequence of detailed, stratified, multistage, and ongoing surveys carried out to evaluate the dietary habits and health condition of a representative segment of individuals residing in the US community. Each year, NHANES assesses about 5,000 individuals through comprehensive interviews and medical evaluations. The interviews collect a wide range of data, including population characteristics, economic status, dietary intake, and data about health; the medical evaluations involve laboratory analyses and physiological assessments. It was obligatory for all participants to furnish written informed consent preceding their incorporation into the study. Moreover, the survey protocol received thorough ethical evaluation and approval from the NCHS Research Ethics Review Board, thereby ensuring compliance with established ethical standards and guidelines throughout the research process.

### 2.2 Study population

**Figure 1:**
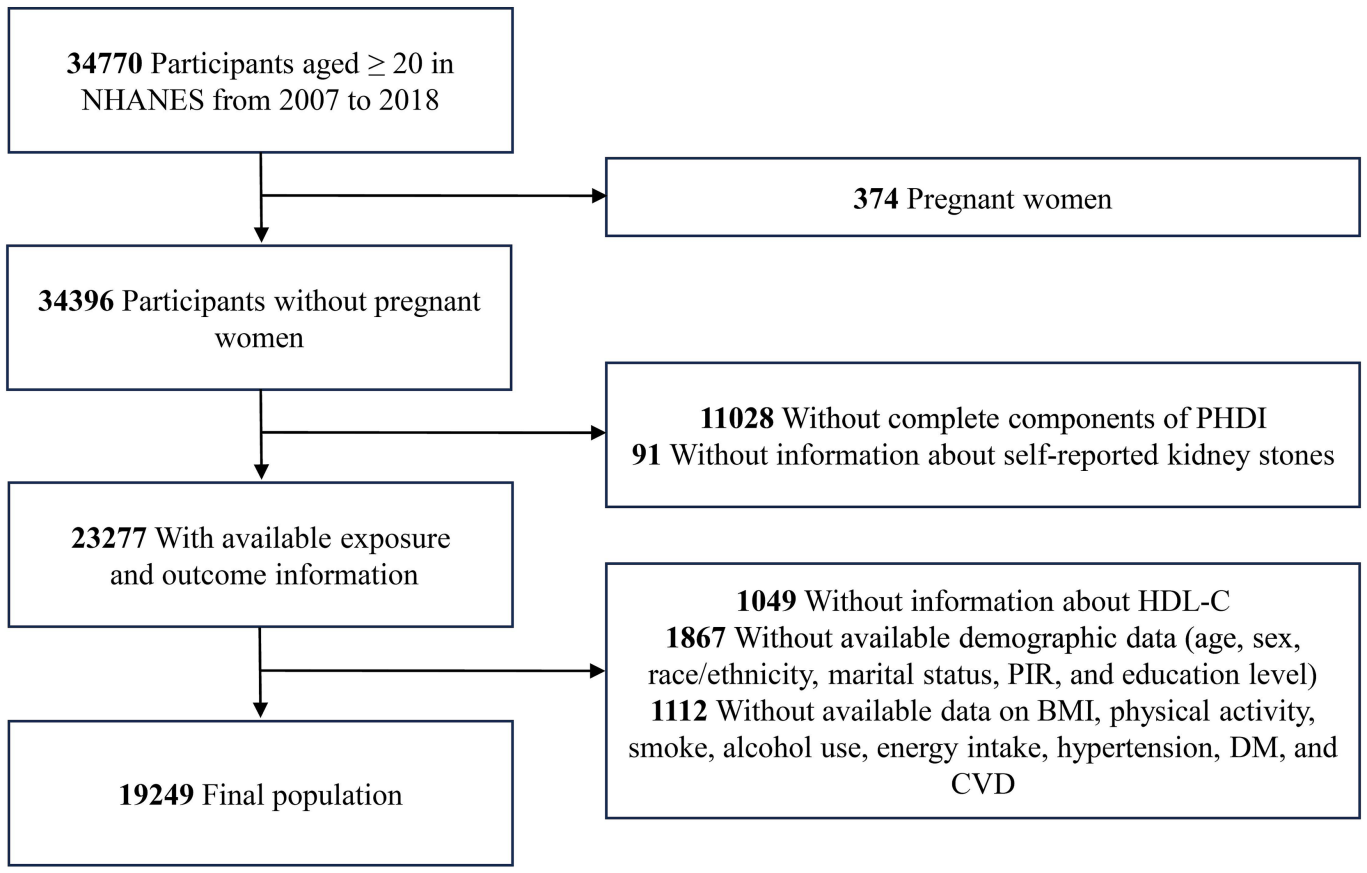
The flow chart of the study. BMI, body mass index; CVD, cardiovascular disease; DM, diabetes mellitus; HDL-C, high-density lipoprotein cholesterol; NHANES, National Health and Nutrition Examination Survey; PHDI, Planetary Health Diet Index; PIR, poverty income ratio.

The study utilized data from six NHANES survey cycles, involving 34,770 participants aged 20 years or over. Exclusion criteria included pregnant women (*n* = 374), participants without the requisite information on PHDI (*n* = 11,028), self-reported kidney stones (*n* = 91), HDL-C (*n* = 1,049), and participants with missing data on demographics variables (*n* = 1,867), as well as participants with unavailable data on body mass index (BMI), physical activity, smoke, alcohol use, energy intake, hypertension, DM, and CVD. The study ultimately included 19,249 participants (Figure 1).

### 2.3 Measurement of PHDI

We calculated PHDI based on two 24-h dietary recall reports. The PHDI includes 15 food components with scores ranging from 0 to 140. Supplementary Table 1 provides a comprehensive overview of the components and scoring criteria for the PHDI, and the reliability of the scoring system has been validated in previous studies (10–12).

### 2.4 Diagnosis of kidney stones

Kidney stone diagnoses were based on self-reports. The participants were posed the question, “Have you ever had kidney stones?,” with answers affirming this condition being categorized as positive. The validity of self-reported diagnoses of kidney stones has hitherto been established in preceding studies (see Supplementary Method 1) (23).

### 2.5 Measurement of HDL-C

The measurement of HDL-C was achieved by a direct immunoassay (see Supplementary Method 2). Detailed laboratory methods are available on the NHANES website.

### 2.6 Covariates

The covariates employed in our study were broadly divided into four primary categories: demographic variables (age, sex, race/ethnicity, marital status, poverty income ratio (PIR), and education level); behavioral and lifestyle variables (physical activity, smoke, and alcohol use); body measurements (BMI and energy intake); and history of medical conditions (hypertension, DM, and CVD). Comprehensive elucidation of the covariates can be accessed from Supplementary Method 3.

### 2.7 Statistical analysis

In view of the complex sampling design, appropriate weights were applied following the guidelines for NHANES analyses. For continuous variables, the Wilcoxon rank-sum test for complex survey samples was used, whereas the chi-squared test with Rao and Scott’s second-order correction was employed for categorical variables. The analysis of participant characteristics was conducted based on PHDI quintiles. Continuous variables were reported as mean values accompanied by standard errors (SE), while categorical variables were displayed as numbers and percentages.

The study’s primary objective was to examine the association between PHDI and self-reported kidney stones; secondary objectives included evaluating the relationship between HDL-C and self-reported kidney stones and determining whether HDL-C mediates the association between PHDI and self-reported kidney stones. The PHDI was entered into the regression analyses in two different forms: a continuous variable, with increments of 10 points, and a categorical variable, with quintiles. We used a weighted multivariable logistic regression model to analyze the link between PHDI and its components with self-reported kidney stones, and the correlation between HDL-C and self-reported kidney stones. To minimize potential bias, we employed propensity score matching (PSM) (1:1) to create a control group and conducted weighted multivariable logistic regression on the matched data to assess the relationship between PHDI and self-reported kidney stones. The relationship between PHDI and HDL-C was assessed using a weighted multivariable linear model. Three distinct models were analyzed in our study. Model 1 was the unadjusted crude model with no covariate adjustments. Model 2 involved adjustments for age, sex, race/ethnicity, marital status, PIR, and education level. Model 3 extended the adjustments from Model 2 by incorporating additional confounders, including BMI, physical activity, smoke, alcohol use, energy intake, hypertension, DM, and CVD. We utilized a restricted cubic spline (RCS) analysis to elucidate the dose–response association, investigating the potential nonlinear relationship between PHDI and self-reported kidney stone risk.

In pursuit of elucidating the mediating function of HDL-C in the relationship between PHDI and self-reported kidney stones, we conducted mediation analyses using the R package “mediation.” Stratified analyses were conducted by age, sex, race/ethnicity, marital status, educational level, smoke, alcohol use, hypertension, DM, and CVD. To assess interaction effects, we added an interaction term to the regression model to determine how the influence of one factor on the outcome changes depending on the level of another factor. In the sensitivity analysis, we utilized the R package “mice” to impute missing covariate data by chained-equation multivariate estimation, followed by the reassessment of the connection between PHDI and self-reported kidney stone risk.

All statistical analyses utilized R (version 4.4.1), supplemented by the survey package (version 4.1–1), and a *p*-value of less than 0.05 on a two-sided test was indicative of statistical significance.

## 3 Results

### 3.1 Baseline characteristics

Table 1 offers a comprehensive presentation of the baseline characteristics of 19,249 US adults (weighted population = 169,992,653) grouped by PHDI quintiles. The weighted mean age was 48.27 (SE = 0.30) years, and female participants accounted for 52.41% of the total. In comparison with participants in the bottom PHDI quintile, those in the top quintile were inclined to be of advanced age, female, married, have a higher PIR, possess an education level above high school, have a lower BMI, engage in physical activity actively, never smoke or drink alcohol, have lower energy intake, not have hypertension, have higher HDL-C levels, and not have self-reported kidney stones. After PSM, no significant differences in baseline characteristics were observed between the groups with and without self-reported kidney stones. Each group comprised 1,923 cases (Supplementary Table 2).

**Table 1:** Characteristics of participants in the NHANES 2007–2018 cycles.

Characteristic	PHDI quintile						
	Overall (19,249)	Q1 (3,850)	Q2 (3,850)	Q3 (3,849)	Q4 (3,850)	Q5 (3,850)	*P* value
Weighted population	169,992,653	32,567,152	34,147,456	35,487,603	33,573,419	34,217,023	
Age, mean (SE), years	48.27(0.30)	44.14 (0.34)	46.44 (0.44)	48.60 (0.50)	50.55 (0.51)	51.42 (0.53)	<0.001
Age group, no. (%)							<0.001
20–39	5,873 (34.57)	1,518 (43.77)	1,316 (37.93)	1,138 (33.36)	1,037 (30.65)	864 (27.58)	
40–59	6,443 (37.61)	1,298 (36.72)	1,287 (37.88)	1,288 (38.82)	1,242 (36.22)	1,328 (38.27)	
≥60	6,933 (27.82)	1,034 (19.51)	1,247 (24.19)	1,423 (27.83)	1,571 (33.13)	1,658 (34.15)	
Sex, no. (%)							<0.001
Female	10,095 (52.41)	1,641 (42.73)	1,950 (49.50)	2,043 (53.31)	2,190 (56.95)	2,271 (59.12)	
Male	9,154 (47.59)	2,209 (57.27)	1,900 (50.50)	1,806 (46.69)	1,660 (43.05)	1,579 (40.88)	
Race/ethnicity, no. (%)							<0.001
Mexican American	2,694 (8.13)	386 (6.26)	512 (8.68)	603 (8.68)	625 (8.92)	568 (8.02)	
Non-Hispanic Black	3,748 (9.57)	1,025 (14.43)	924 (12.16)	734 (9.08)	568 (7.02)	497 (5.38)	
Non-Hispanic White	8,891 (69.55)	1,938 (69.49)	1,800 (68.41)	1,804 (71.40)	1,740 (69.26)	1,609 (69.10)	
Other Hispanic	1,875 (5.25)	291 (4.97)	355 (5.18)	405 (5.22)	459 (6.07)	365 (4.83)	
Other Race	2,041 (7.50)	210 (4.86)	259 (5.57)	303 (5.62)	458 (8.74)	811 (12.67)	
Marital status, no. (%)							<0.001
Married	10,313 (56.91)	1,824 (49.41)	1,911 (52.62)	2,019 (56.97)	2,201 (61.78)	2,358 (63.49)	
Never married	3,224 (17.63)	821 (22.05)	731 (19.47)	650 (17.85)	522 (14.15)	500 (14.77)	
Living with partner	1,447 (7.62)	396 (10.11)	328 (8.91)	291 (7.02)	239 (6.27)	193 (5.91)	
Other	4,265 (17.84)	809 (18.43)	880 (19.00)	889 (18.16)	888 (17.81)	799 (15.83)	
PIR, Mean (SE)	3.09 (0.04)	2.63 (0.05)	2.85 (0.05)	3.11 (0.05)	3.31 (0.05)	3.52 (0.05)	<0.001
Education level, no. (%)							<0.001
Less than high school	4,003 (13.08)	987 (18.30)	861 (15.03)	786 (11.80)	776 (12.10)	593 (8.45)	
High school or equivalent	4,382 (22.69)	1,134 (31.08)	1,015 (25.38)	940 (24.67)	752 (19.74)	541 (12.88)	
Above high school	10,864 (64.23)	1,729 (50.62)	1,974 (59.58)	2,123 (63.53)	2,322 (68.17)	2,716 (78.66)	
BMI, mean (SE), kg/m^2^	29.19 (0.11)	30.19 (0.19)	29.90 (0.17)	29.55 (0.16)	28.69 (0.18)	27.63 (0.17)	<0.001
Physical activity, min/wk., no. (%)							<0.001
None (inactive)	4,940 (20.66)	1,079 (23.53)	1,115 (24.17)	1,004 (20.71)	976 (20.05)	766 (14.98)	
0 to <150 (insufficiently active)	2,638 (13.48)	495 (12.98)	533 (14.06)	522 (14.39)	559 (15.05)	529 (10.90)	
≥150 (active)	11,671 (65.86)	2,276 (63.50)	2,202 (61.76)	2,323 (64.90)	2,315 (64.89)	2,555 (74.13)	
Smoke, no. (%)							<0.001
Never	10,778 (56.68)	1,726 (46.08)	1,974 (53.85)	2,163 (56.54)	2,358 (61.99)	2,557 (64.53)	
Former	4,955 (25.71)	849 (22.30)	929 (22.90)	1,056 (27.23)	1,082 (27.01)	1,039 (28.89)	
Now	3,516 (17.61)	1,275 (31.62)	947 (23.25)	630 (16.24)	410 (11.00)	254 (6.58)	
Alcohol use, no. (%)							<0.001
Never	2,518 (10.39)	376 (7.89)	482 (11.40)	486 (10.27)	530 (10.01)	644 (12.27)	
Former	3,608 (15.28)	844 (19.04)	732 (15.78)	709 (14.42)	703 (14.24)	620 (13.10)	
Now	13,123 (74.33)	2,630 (73.06)	2,636 (72.82)	2,654 (75.31)	2,617 (75.75)	2,586 (74.63)	
Energy intake, mean (SE), kcal/d	2,013.09 (6.75)	2,128.62 (13.46)	2,015.22 (15.01)	2,005.52 (16.21)	1,963.70 (15.12)	1,957.31 (17.68)	<0.001
Hypertension, no. (%)							0.02
No	10,755 (61.70)	2,230 (63.27)	2,157 (62.55)	2,108 (60.61)	2,072 (58.37)	2,188 (63.74)	
Yes	8,494 (38.30)	1,620 (36.73)	1,693 (37.45)	1,741 (39.39)	1,778 (41.63)	1,662 (36.26)	
DM, no. (%)							0.77
No	15,497 (85.50)	3,190 (86.25)	3,127 (85.92)	3,040 (84.97)	3,065 (85.15)	3,075 (85.29)	
Yes	3,752 (14.50)	660 (13.75)	723 (14.08)	809 (15.03)	785 (14.85)	775 (14.71)	
CVD, no. (%)							0.25
No	17,066 (91.19)	3,412 (91.89)	3,394 (91.27)	3,395 (90.35)	3,392 (90.60)	3,473 (91.87)	
Yes	2,183 (8.81)	438 (8.11)	456 (8.73)	454 (9.65)	458 (9.40)	377 (8.13)	
HDL-C, mean (SE), mg/dL	53.39 (0.24)	49.01 (0.32)	51.36 (0.34)	53.88 (0.39)	54.97 (0.50)	57.54 (0.44)	<0.001
Self-reported kidney stones, no. (%)							0.03
No	17,326 (90.02)	3,439 (89.19)	3,418 (88.20)	3,466 (90.59)	3,486 (90.97)	3,517 (91.13)	
Yes	1,923 (9.98)	411 (10.81)	432 (11.80)	383 (9.41)	364 (9.03)	333 (8.87)	

BMI, body mass index; CVD, cardiovascular disease; DM, diabetes mellitus; HDL-C, high-density lipoprotein cholesterol; NHANES, National Health and Nutrition Examination Survey; PHDI, Planetary Health Diet Index; PIR, poverty income ratio; Q, quintile; SE, standard error. All means and SEs for continuous variables and percentages for categorical variables were weighted.

### 3.2 Association between PHDI and self-reported kidney stones

**Figure 2:**
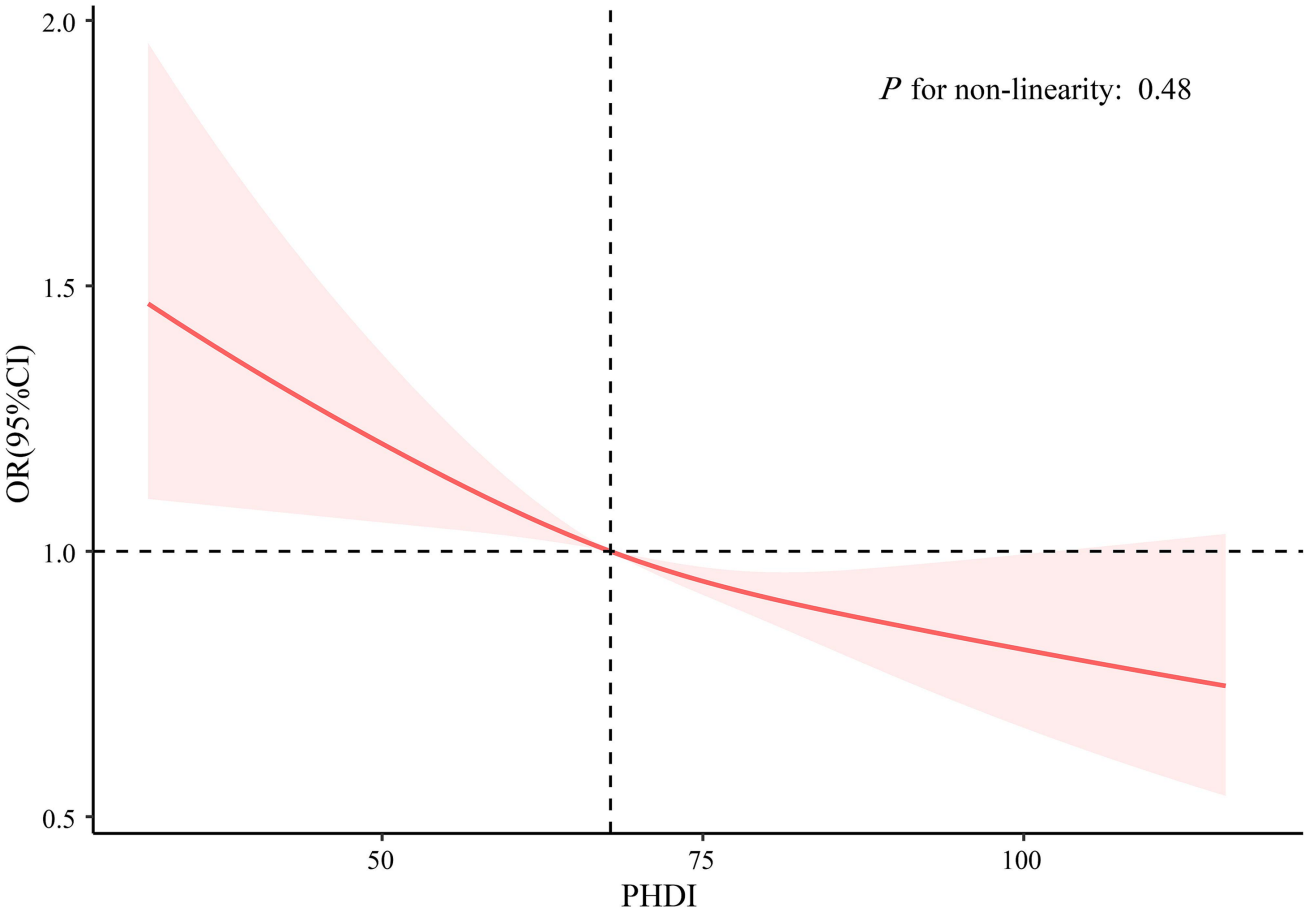
Association between PHDI and self-reported kidney stones using restricted cubic splines regression. The model was adjusted for age, sex, race/ethnicity, marital status, PIR, education level, BMI, physical activity, smoke, alcohol use, energy intake, hypertension, DM, and CVD. Abbreviations: BMI, body mass index; CI, Confidence Interval; CVD, cardiovascular disease; DM, diabetes mellitus; OR, Odd Ratio; PHDI, Planetary Health Diet Index; PIR, poverty income ratio.

As elucidated in Table 2, there exists a demonstrable correlation between PHDI and the incidence of self-reported kidney stones. The findings in Model 3 indicated an 8% decrease in the likelihood of developing self-reported kidney stones for every 10-point elevation in PHDI (OR, 0.92; 95% CI, 0.87–0.97). Besides, participants within the uppermost PHDI quintile exhibited a 25% decreased risk of self-reported kidney stones in comparison to those within the nethermost quintile (OR, 0.75; 95% CI, 0.58–0.96). After PSM, the association between PHDI and self-reported kidney stones remained largely unchanged (Table 2). To further examine the dose–response relationship, an RCS analysis was performed, which revealed an inverse linear relationship between PHDI and the presence of self-reported kidney stones (*P* for non-linearity: 0.48; Figure 2). Supplementary Table 3 presents the link between PHDI components and self-reported kidney stone risk. Whole grains, non-starchy vegetables, whole fruits, dairy, and added sugar were significant in the fully adjusted model.

**Table 2:** Association between PHDI and self-reported kidney stones.

PSM status	Variable	Model 1^a^	*P*-value	Model 2^b^	*P*-value	Model 3^c^	*P*-value
		OR (95%CI)		OR (95%CI)		OR (95%CI)	
Before PSM	PHDI score^d^	0.94 (0.89, 0.99)	0.02	0.90 (0.85, 0.95)	<0.001	0.92 (0.87, 0.97)	0.005
	Quintile						
	Q1	1[Reference]		1[Reference]		1[Reference]	
	Q2	1.10 (0.87, 1.40)	0.41	1.05 (0.82, 1.34)	0.7	1.08 (0.84, 1.38)	0.56
	Q3	0.86 (0.69, 1.06)	0.14	0.76 (0.61, 0.95)	0.02	0.78 (0.63, 0.98)	0.03
	Q4	0.82 (0.67, 1.00)	0.06	0.70 (0.56, 0.87)	0.001	0.74 (0.60, 0.92)	0.01
	Q5	0.80 (0.64, 1.01)	0.06	0.67 (0.53, 0.86)	0.002	0.75 (0.58, 0.96)	0.02
	Trend test		0.004		<0.001		<0.001
After PSM	PHDI score^d^	0.94 (0.90, 0.99)	0.01	0.93 (0.88, 0.98)	0.004	0.93 (0.88, 0.98)	0.01
	Quintile						
	Q1	1[Reference]		1[Reference]		1[Reference]	
	Q2	1.06 (0.87, 1.29)	0.56	1.05 (0.86, 1.28)	0.65	1.05 (0.86, 1.28)	0.64
	Q3	0.89 (0.73, 1.09)	0.27	0.88 (0.71, 1.07)	0.20	0.88 (0.72, 1.08)	0.23
	Q4	0.83 (0.68, 1.01)	0.06	0.81 (0.65, 0.99)	0.04	0.81 (0.66, 0.99)	0.04
	Q5	0.85 (0.69, 1.04)	0.11	0.81 (0.65, 1.00)	0.05	0.81 (0.65, 1.01)	0.06
	Trend test		0.01		0.005		0.01

BMI, body mass index; CI, Confidence Interval; CVD, cardiovascular disease; DM, diabetes mellitus; OR, Odd Ratio; PHDI, Planetary Health Diet Index; PIR, poverty income ratio; PSM, Propensity Score Matching; Q, quintile.

^a^Crude model.

^b^Adjusted for age, sex, race/ethnicity, marital status, PIR, education level.

^c^Adjusted for age, sex, race/ethnicity, marital status, PIR, education level, BMI, physical activity, smoke, alcohol use, energy intake, hypertension, DM, and CVD.

^d^PHDI score was entered as a continuous variable per 10 points increase.

### 3.3 Mediation analyses

**Figure 3:**
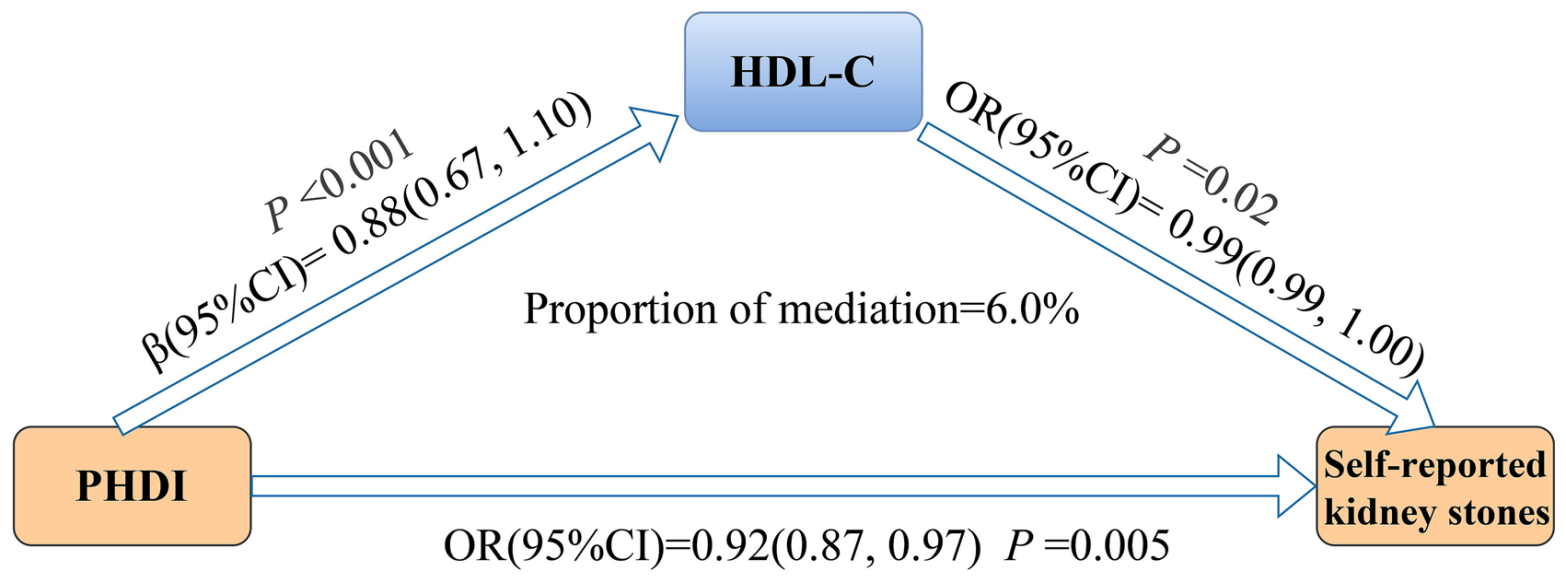
Mediation analysis of HDL-C in the association between PHDI and self-reported kidney stones. The model was adjusted for age, sex, race/ethnicity, marital status, PIR, education level, BMI, physical activity, smoke, alcohol use, energy intake, hypertension, DM, and CVD. Abbreviations: BMI, body mass index; CI, Confidence Interval; CVD, cardiovascular disease; DM, diabetes mellitus; HDL-C, high-density lipoprotein cholesterol; OR, Odd Ratio; PHDI, Planetary Health Diet Index; PIR, poverty income ratio.

As shown in Supplementary Table 4, each 10-point rise in PHDI was related to a 0.88 mg/dL increase in HDL-C (*β*, 0.88; 95% CI, 0.67–1.10). Additionally, HDL-C was inversely associated with self-reported kidney stones (OR, 0.99; 95% CI, 0.99–1.00). Figure 3 illustrates that HDL-C mediated 6.0% of the relationship between PHDI and self-reported kidney stones. Detailed mediating effects are provided in Supplementary Table 5.

### 3.4 Subgroup and sensitivity analyses

**Figure 4:**
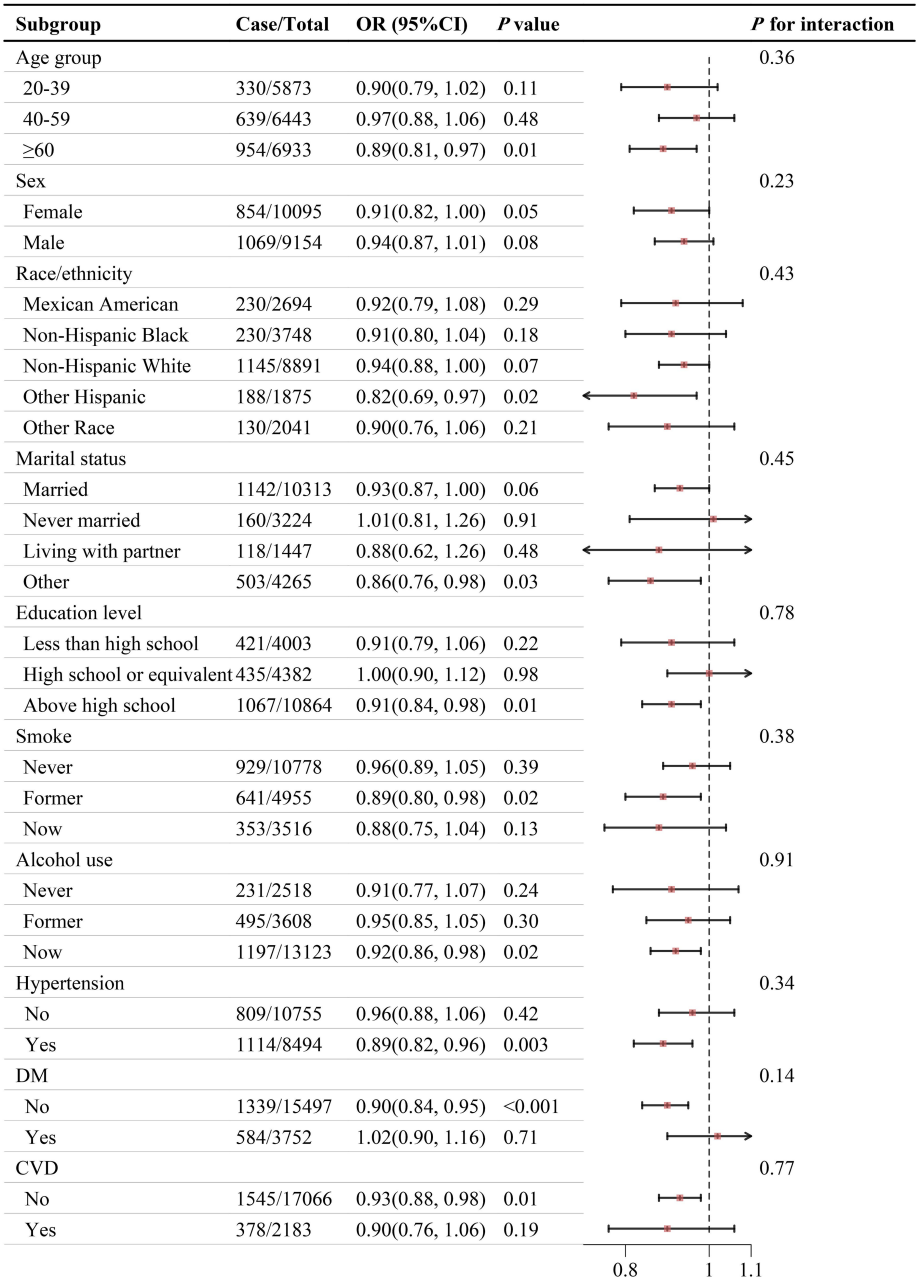
Association between PHDI and self-reported kidney stones in different subgroups. The model was adjusted for age, sex, race/ethnicity, marital status, PIR, education level, BMI, physical activity, smoke, alcohol use, energy intake, hypertension, DM, and CVD. Abbreviations: BMI, body mass index; CI, Confidence Interval; CVD, cardiovascular disease; DM, diabetes mellitus; OR, Odd Ratio; PHDI, Planetary Health Diet Index; PIR, poverty income ratio.

Figure 4 shows no significant interaction between PHDI and self-reported kidney stone risk across different subgroups (*P* for interaction > 0.05). Sensitivity analyses suggested that the relationship between PHDI and self-reported kidney stones remained consistent after multiple imputations for missing covariates (Supplementary Table 6).

## 4 Discussion

This study was the earliest extensive cross-sectional analysis to explore the potential correlation between PHDI and self-reported kidney stones. PHDI was found to reduce self-reported kidney stone risk linearly. PHDI was positively correlated with HDL-C, which was negatively related to self-reported kidney stones. Mediation analysis indicated that HDL-C mediated 6.0% of the relationship between PHDI and self-reported kidney stones.

We observed significant heterogeneity in baseline characteristics across PHDI quintiles. The high-PHDI group exhibited not only better dietary quality but also lower BMI, higher physical activity levels, and reduced smoking and drinking rates. Additionally, this group showed a significantly lower prevalence of hypertension compared to the low-PHDI group. These confounding factors may collectively influence self-reported kidney stone risk through mechanisms such as weight control and changes in urinary biochemistry (24, 25). Given these baseline differences, caution is warranted in interpreting the results. To account for confounding, we constructed weighted multivariable logistic regression models that incorporated demographic factors, lifestyle variables, and comorbidities. Additionally, we employed PSM to balance covariates. The results from both approaches were highly consistent, suggesting that the negative association between PHDI and self-reported kidney stone risk remains independent even after controlling for confounding. In addition, subgroup analyses confirmed that the association between PHDI and self-reported kidney stones held across various characteristics, including age, gender, and ethnicity, suggesting that our results could be extrapolated to a broader US adult population. Besides, sensitivity analyses further consolidated the robustness of the results, with PHDI consistently linked with a diminished susceptibility to self-reported kidney stones.

Several studies have investigated the relationship between other dietary patterns and kidney stones. A large longitudinal study of three distinct cohorts by Rodriguez et al. (26) concluded that following the Mediterranean diet diminished kidney stone prevalence. In the Seguimiento Universidad de Navarra (SUN) Cohort, individuals in the highest Mediterranean dietary pattern score group (7–9 points) had a 36% reduced risk of kidney stones compared to those in the lowest group (0–3 points) (27). Similarly, the Dietary Approaches to Stop Hypertension (DASH) diet was found to curtail urinary calcium oxalate supersaturation by augmenting urine volume, pH, citrate and magnesium excretion, thereby lowering kidney stone risk (28, 29). In the Health Professionals Follow-up Study, participants in the highest quintile of DASH scores had a 45% lower risk of kidney stones compared to those in the lowest quintile (30). The Healthy Eating Index-2015 (HEI-2015) was also observed to prevent kidney stones, specifically, compared to the lowest quartile, the highest quartile of the HEI-2015 was associated with a roughly 30% reduction in kidney stone prevalence among US adults (31). Like these plant-based diets, adopting PHDI was a preventative strategy for kidney stones. The highest quintile of PHDI was associated with a 25% reduction in self-reported kidney stone risk compared to the lowest quintile. However, most dietary patterns neglect their environmental consequences, whereas the PHDI offers substantial ecological benefits. The Earth’s environment currently faces severe threats—frequent extreme weather events driven by global warming, sea-level rise from glacial melt, rapid biodiversity loss, freshwater scarcity, and escalating pollution (32, 33). Different diets exert varying environmental pressures: meat-based regimens demand extensive land and water resources and generate high greenhouse-gas emissions, thereby accelerating climate change, ecosystem degradation, and biodiversity decline (34, 35). In contrast, the PHDI emphasizes plant-based foods and limits red meat and processed items, reducing greenhouse gas emissions and conserving land and water, which alleviates environmental stress and supports ecosystem preservation (36, 37). Given these considerations, the PHDI not only mitigates present environmental challenges but also promotes sustainable development. Beyond reducing kidney stone risk, previous studies have shown that PHDI was associated with numerous health benefits, including improved cardiovascular health (14), reduced mortality (38), and lower type 2 diabetes risk (39). For individuals at high risk of kidney stones, adopting PHDI not only provides targeted prevention but also offers extra cardiovascular and metabolic benefits. Additionally, this dietary pattern supports environmental sustainability, making it highly relevant for both clinical disease prevention and public health.

Analysis of PHDI components revealed that increased intakes of whole grains, non-starchy vegetables, and whole fruits, along with decreased intakes of added sugars, were significantly related to a reduced risk of self-reported kidney stones in the fully adjusted model, which aligns with PHDI recommendations. Shan et al. (40) revealed that higher whole grain consumption reduced the risk of hospitalized kidney stones. This may be partly attributed to the high phytate content in whole grain bran, which boosts urinary phytate excretion and inhibits calcium oxalate crystal formation (41, 42). Besides, whole grains are a key source of dietary potassium, which increases urinary citrate excretion, a major inhibitor of kidney stones (43). Similarly, vegetables and fruits can also increase urinary citrate, which both inhibits calcium crystal formation and prevents the aggregation of existing calcium oxalate crystals (44, 45). They may also reduce stone formation by increasing urine volume, urinary pH, and the excretion of potassium and magnesium (46). As for added sugar, a study grounded in NHANES data found that a heightened proportion of energy intake derived from added sugar corresponded to an elevated prevalence of kidney stones (47), while another study similarly linked added sugar intake to a high kidney stone risk (48). Fructose, which is one type of added sugar, may lead to stone formation by altering urinary pH, affecting uric acid metabolism, decreasing urinary citrate excretion, increasing urinary oxalate levels, and reducing urinary magnesium excretion (49, 50).

Interestingly, the PHDI recommends a dairy intake of no more than 250 g/d, yet our study found a beneficial impact of increased dairy intake on self-reported kidney stone prevention. This aligned with a prospective cohort study on Mediterranean diets, which also found that increased dairy intake reduced kidney stone prevalence (27). Moreover, Ferraro et al. (51) demonstrated that the correlation between protein and kidney stones varied by protein type, with dairy protein inversely related to kidney stone risk in the Nurses’ Health Study II. Their study also indicated that dairy protein reduced urinary concentrations of oxalate and uric acid and concurrently stimulated urinary citrate excretion (51). In addition, certain fatty acids in dairy products can contribute to the suppression of kidney stones, for instance, polyunsaturated fatty acids may protect against the deposition of calcium oxalate stones (52) and mitigate renal tubular damage induced by kidney stones (53).

Consistent with our findings, Frank et al. (54) discovered that one standard deviation augmentation in PHDI raised HDL-C by 1.9 mg/dL, based on data from the NHANES database. Analogous results were observed by Morcel et al. (21), who reported a positive association between PHDI and HDL-C in European adolescents. The mechanism behind PHDI-induced HDL-C elevation remains unclear, but one possible explanation is that plant-based PHDI increases dietary fiber intake, which is fermented by intestinal bacteria to produce short-chain fatty acids. These short-chain fatty acids can activate peroxisome proliferator-activated receptor-γ (55), which upregulates the expression of the ABCA1 gene, leading to an increase in ABCA1 protein that facilitates the transport of intracellular cholesterol into HDL (56, 57), thereby resulting in elevated HDL-C levels. However, two studies in Brazilian populations found no association between PHDI and HDL-C (58, 59), possibly due to differences in sample sizes, study populations, or PHDI calculation methods. The method used in this study to calculate PHDI has been validated in prior research (10–12).

Additionally, our study identified a negative correlation between HDL-C and self-reported kidney stones, consistent with previous research (22, 60, 61). Although the precise pathway by which HDL-C modulates kidney stone formation remains undefined, elucidating this mechanism warrants further investigation. We believe that the possible mechanism is as follows: Calcium oxalate stones, the most prevalent form, are closely linked to Randall’s plaque, which are calcium phosphate deposits in the renal papillae interstitium (62). These plaques provide a surface for the accumulation of calcium oxalate crystals, facilitating stone attachment (8, 62). This process was thought to be influenced by oxidative stress and inflammation (63). Moreover, HDL-C was found to reduce oxidative stress by promoting nitric oxide production and inhibiting reactive oxygen species production (64). It was also found to be associated with lowered levels of inflammatory markers, encompassing C-reactive protein and tumor necrosis factor-α (65). Thus, HDL-C may decrease Randall’s plaque formation and calcium salt deposition by diminishing oxidative stress and inflammation, thereby lowering kidney stone risk. This study is the first to demonstrate that HDL-C may mediate the relationship between PHDI and self-reported kidney stone formation, thereby filling a critical gap in the literature and providing novel evidence for the “PHDI – HDL-C – kidney stone” pathway. Mediation analysis suggests HDL-C may partially mediate the relationship between PHDI and self-reported kidney stone risk; however, this association does not imply a direct causal link. One possible explanation is that elevated HDL-C levels could reflect a healthy dietary pattern (e.g., higher fiber intake and increased consumption of fruits and vegetables) rather than serve as a causal factor. Previous studies have shown that such dietary patterns can reduce kidney stone risk through mechanisms like increased urine volume, citrate and magnesium excretion, reduced dietary acid load, and decreased oxidative stress (66), some of which may operate independently of HDL-C. Thus, future research should employ more detailed dietary assessments and randomized controlled trials to further clarify the causal role of HDL-C in this pathway. Besides, given the observational design of this study, caution is warranted in interpreting the mediating effects of the mediator.

In addition to the HDL-C pathway, our study found that individuals with higher PHDI scores tended to have lower BMI and better blood pressure control, suggesting other potential mechanisms through which PHDI may reduce kidney stone risk. PHDI supports weight management by optimizing dietary composition (67), and lower BMI has been associated with reduced urinary oxalate concentration, thereby decreasing kidney stone risk (68). Furthermore, PHDI improves vascular function and endothelial health by reducing sodium intake and increasing potassium and fiber consumption, which helps lower hypertension prevalence (69). Hypertension alters urinary composition, promoting calcium reabsorption and elevating urinary calcium levels, thus increasing stone risk (70). Therefore, PHDI may indirectly influence urinary composition by improving weight and blood pressure, thereby reducing kidney stone risk. These mechanisms provide a theoretical framework for understanding the link between PHDI and kidney stones, though further research is needed to explore additional pathways.

However, this study is subject to some limitations. First, PHDI is based on short-term intake data from 24-h dietary recalls, which may not fully capture long-term dietary patterns and are subject to measurement error. Although dietary questionnaires are susceptible to recall bias, the use of repeated 24-h recalls improves the reliability of the dietary assessment. Second, kidney stone outcomes are self-reported, which may be subject to underreporting or misreporting, potentially leading to biases. For example, undiagnosed asymptomatic cases may be underreported, resulting in an underestimation of prevalence. Moreover, the precise timing of self-reported kidney stone diagnosis is unclear, and variations exist among patients diagnosed at different times. For example, a patient diagnosed 20 years ago and stone-free since may differ from one with recurrent stones in the past 3 years. Third, it is incapable of establishing causality between PHDI and self-reported kidney stones because of the cross-sectional design. Fourth, this study is based on US population data, and the relationship between PHDI and kidney stones may differ in other countries or dietary contexts. Therefore, conclusions should be extended with caution, and further research is needed to confirm this association in diverse populations. Fifth, the NHANES database is deficient in available data concerning the composition of kidney stones, warranting future research to explore whether the link between PHDI and kidney stones is influenced by stone composition and the underlying mechanisms. Notwithstanding the aforementioned limitations, our study was innovative in its identification of a negative correlation between PHDI and self-reported kidney stones, with HDL-C functioning as a mediator, which is crucial for both kidney stone prevention and planetary sustainability.

## 5 Conclusion

This large cross-sectional NHANES study ascertained that adhering to the PHDI was related to a reduced vulnerability to self-reported kidney stones, with HDL-C partially mediating this negative relationship. Promoting PHDI is of pivotal importance for both kidney stone prevention and environmental sustainability.

## Data Availability

Publicly available datasets were analyzed in this study. This data can be found: https://www.cdc.gov/nchs/nhanes.
