# Supplementary material for: Planetary Health Diet Index and self-reported kidney stones in US adults: mediating role of high-density lipoprotein cholesterol
Source: Front Nutr. 2025 Nov 10;12:1609626. doi: 10.3389/fnut.2025.1609626 (PMC12640945; doi:10.3389/fnut.2025.1609626)
Supplement: Supplementary file 1 [file Table_1.docx]

**Supplementary Material Files**

**Supplementary Text**

Supplementary Method 1. Validating Self-Reported Kidney Stones in NHANES: Evidence from Prospective Cohorts.

Supplementary Method 2. Assessment of HDL-C.

Supplementary Method 3. Definition and Grouping of Covariates.

**Supplementary Table**

Supplementary Table 1. The Planetary Health Diet Index components and criteria for scoring.

Supplementary Table 2. Characteristics of participants in the NHANES 2007-2018 cycles after propensity score matching.

Supplementary Table 3. Association between PHDI component score and self-reported kidney stones.

Supplementary Table 4. Associations between HDL-C and PHDI and self-reported kidney stones.

Supplementary Table 5. The mediating effect of HDL-C in the association between PHDI and self-reported kidney stones.

Supplementary Table 6. Association between PHDI and self-reported kidney stones with multiple imputation analysis.

**Supplementary Method 1. Validating Self-Reported Kidney Stones in NHANES: Evidence from Prospective Cohorts.**

In large-scale epidemiological surveys such as NHANES, efficient and cost-effective data collection is essential. Self-reported kidney-stone history meets these criteria and has demonstrated high validity: medical-record review of 60 men in the Health Professionals Follow-up Study confirmed 97 % of self-reported events (1), and review of 90 women in Nurses’ Health Study I corroborated 98 % (2). Although NHANES’s de-identified design precludes individual validation, numerous NHANES-based investigations have successfully employed self-report to estimate nephrolithiasis prevalence (3–5). Therefore, self-reported kidney stones offer a rigorous and feasible method for identifying affected participants in NHANES.

References:

1. En T, Mj S, Gc C. Obesity, weight gain, and the risk of kidney stones. *JAMA* (2005) 293: doi: 10.1001/jama.293.4.455

2. Gc C, Wc W, Fe S, D S, Mj S. Comparison of dietary calcium with supplemental calcium and other nutrients as factors affecting the risk for kidney stones in women. *Annals of internal medicine* (1997) 126: doi: 10.7326/0003-4819-126-7-199704010-00001

3. Cd S, Ac S, Jm H, Cs S. Prevalence of kidney stones in the United States. *European urology* (2012) 62: doi: 10.1016/j.eururo.2012.03.052

4. G L, F Z, W R, Y P, W W. Association between novel anthropometric indices and prevalence of kidney stones in US adults. *World journal of urology* (2023) 41: doi: 10.1007/s00345-023-04582-x

5. M W, M L, Y Z, J W, M G, F H, H C, Z Z. Serum HDL partially mediates the association between exposure to volatile organic compounds and kidney stones: A nationally representative cross-sectional study from NHANES. *The Science of the total environment* (2024) 907: doi: 10.1016/j.scitotenv.2023.167915

**Supplementary Method 2. Assessment of HDL-C.**

HDL-C was measured by direct immunoassay at the University of Minnesota using Roche’s modular P and Cobas 6000 analyzers. In each serum sample, a magnesium sulfate/dextran sulfate reagent selectively complexes non-HDL lipoproteins, leaving only HDL available for reaction. Next, cholesterol esters in HDL are hydrolyzed to free cholesterol by PEG-cholesterol esterase, which is then oxidized by PEG-cholesterol oxidase to generate hydrogen peroxide. Finally, peroxidase catalyzes the reaction of hydrogen peroxide with 4-aminoantipyrine and HSDA, producing a purple-blue dye whose intensity is measured photometrically at 600 nm (700 nm secondary).

**Supplementary Method 3. Definition and Grouping of Covariates.**

Age was classified into three groups: <40, 40-59, and ≥60 years old. Self-reported race/ethnicity included Mexican American, non-Hispanic black, non-Hispanic white, other Hispanic, or other. Marital status encompassed married, never married, living with partner, and other, which comprised widowed, divorced, or separated individuals. Poverty income ratio (PIR) was calculated by dividing family (or individual) income by poverty guideline. Educational level was divided into three groups: less than high school, high school or equivalent, and above high school. BMI was calculated as weight in kilograms divided by height in meters squared. Physical activity was categorized into three groups: inactive (0 minutes per week), insufficiently active (0 to <150 minutes per week), and active (≥150 minutes per week). Smoke was categorized into three groups: never (<100 cigarettes), former (≥100 cigarettes and had quit smoking), and now (≥100 cigarettes and currently smoke). Alcohol use was self-reported and categorized as follows: never (<12 drinks in a lifetime), former (<12 drinks in the past year) and now (≥12 drinks in the past year). Dietary energy intake data were collected through 24-hour dietary recall interviews conducted at the mobile examination center (MEC). Hypertension was diagnosed if systolic blood pressure was ≥140 mmHg or diastolic blood pressure was ≥90 mmHg. The diagnostic criteria for DM encompass a physician’s diagnosis, glycohemoglobin (HbA1c) levels exceeding 6.5%, fasting glucose levels of 7.0 mmol/L or higher, random or two-hour oral glucose tolerance test (OGTT) blood glucose levels of 11.1 mmol/L or above, or the administration of diabetes medication/insulin. The CVD history was self-reported and included previous diagnoses of heart failure, coronary heart disease, angina, heart attack, or stroke.

**Supplementary Table 1. The Planetary Health Diet Index components and criteria for scoring.**

| **Dietary component** | **Min score (0 points)** | **Max score** | **Max score** |
| --- | --- | --- | --- |
| Whole grain | 0 g/d | ≥75 g/d (female)  ≥90 g/d (male) | 10 |
| Non-starchy Vegetable | 0 g/d | > 300 g/d | 10 |
| Whole fruit | 0 g/d | > 200 g/d | 10 |
| Fish and Shellfish | 0 g/d | > 28 g/d | 10 |
| Nuts and Seeds | 0 g/d | > 50 g/d | 10 |
| Added Fat, Unsaturated | < 3.5 % of total energy intake | > 21 % of total energy intake | 10 |
| Non-soy Legume | 0 g/d | > 100 g/d | 5 |
| Soybean | 0 g/d | > 50 g/d | 5 |
| Starchy Vegetable | > 200 g/d | < 50 g/d | 10 |
| Dairy | > 1000 g/d | < 250 g/d | 10 |
| Red and Processed Meat | > 100 g/d | < 14 g/d | 10 |
| Poultry | > 100 g/d | < 29 g/d | 10 |
| Egg | > 120 g/d | < 13 g/d | 10 |
| Added Fat, Saturated | > 10 % of total energy intake | 0 % of total energy intake | 10 |
| Added sugar | > 25 % of total energy intake | < 5 % of total energy intake | 10 |

Food consumption is scored proportionally. For instance, for non-starchy vegetables, the scoring is 0 if 0 grams are consumed daily, and 10 if ≥300 grams are consumed. For someone consuming 150 grams a day, the score is 150/[(300-0)/10] = 5.

**Supplementary Table 2.** **Characteristics of participants in the NHANES 2007-2018 cycles after propensity score matching.**

| **Characteristic** | **Without self-reported kidney stones (N = 1923)** | **With self-reported kidney stones (N = 1923)** | ***P* value** |
| --- | --- | --- | --- |
| Age, Mean (SD), years | 56.93 (16.98) | 56.93 (15.81) | 0.993 |
| Sex, No. (%) |  |  | 0.795 |
| Female | 863 (44.9) | 854 (44.4) |  |
| Male | 1060 (55.1) | 1069 (55.6) |  |
| Race/ethnicity, No. (%) |  |  | 0.808 |
| Mexican American | 225 (11.7) | 230 (12.0) |  |
| Non-Hispanic Black | 233 (12.1) | 230 (12.0) |  |
| Non-Hispanic White | 1144 (59.5) | 1145 (59.5) |  |
| Other Hispanic | 205 (10.7) | 188 (9.8) |  |
| Other Race | 116 (6.0) | 130 (6.8) |  |
| Marital status, No. (%) |  |  | 0.897 |
| Married | 1143 (59.4) | 1142 (59.4) |  |
| Never married | 149 (7.7) | 160 (8.3) |  |
| Living with partner | 115 (6.0) | 118 (6.1) |  |
| Other | 516 (26.8) | 503 (26.2) |  |
| PIR, Mean (SD) | 2.57 (1.62) | 2.58 (1.61) | 0.787 |
| Education level, No. (%) |  |  | 0.684 |
| Less than high school | 440 (22.9) | 421 (21.9) |  |
| High school or equivalent | 418 (21.7) | 435 (22.6) |  |
| Above high school | 1065 (55.4) | 1067 (55.5) |  |
| BMI, mean (SD), kg/m^2^ | 30.57 (7.25) | 30.64 (6.86) | 0.763 |
| Physical activity, min/wk, No. (%) |  |  | 0.057 |
| None (inactive) | 546 (28.4) | 30.64 (6.86) |  |
| 0 to <150 (insufficiently active) | 246 (12.8) | 246 (12.8) |  |
| ≥150 (active) | 1131 (58.8) | 1065 (55.4) |  |
| Smoke, No. (%) |  |  | 0.678 |
| Never | 919 (47.8) | 929 (48.3) |  |
| Former | 665 (34.6) | 641 (33.3) |  |
| Now | 339 (17.6) | 353 (18.4) |  |
| Alcohol use, No. (%) |  |  | 0.560 |
| Never | 247 (12.8) | 231 (12.0) |  |
| Former | 471 (24.5) | 495 (25.7) |  |
| Now | 1205 (62.7) | 1197 (62.2) |  |
| Energy intake, mean (SD), kcal/d | 1961.82 (634.29) | 1954.91 (616.02) | 0.732 |
| Hypertension, No. (%) |  |  | 0.974 |
| No | 811 (42.2) | 809 (42.1) |  |
| Yes | 1112 (57.8) | 1114 (57.9) |  |
| DM, No. (%) |  |  | 0.481 |
| No | 1360 (70.7) | 1339 (69.6) |  |
| Yes | 563 (29.3) | 584 (30.4) |  |
| CVD, No. (%) |  |  | 0.399 |
| No | 1523 (79.2) | 1545 (80.3) |  |
| Yes | 400 (20.8) | 378 (19.7) |  |

Abbreviations: BMI, body mass index; CVD, cardiovascular disease; DM, diabetes mellitus; NHANES, National Health and Nutrition Examination Survey; PIR, poverty income ratio; Q, quintile; SD, Standard Deviation. The Wilcoxon rank sum test compared continuous variables, and Pearson’s Chi-squared test compared categorical variables for intergroup differences.

**Supplementary Table 3. Association between PHDI component score and self-reported kidney stones.**

|  | **Model 1^a^** |  | **Model 2^b^** |  | **Model 3^c^** |  |
| --- | --- | --- | --- | --- | --- | --- |
| **PHDI component score** | **OR(95%CI)** | ***P* value** | **OR(95%CI)** | ***P* value** | **OR(95%CI)** | ***P* value** |
| Whole grain score | 0.88(0.74, 1.04) | 0.13 | 0.69(0.58, 0.83) | <0.001 | 0.74(0.62, 0.89) | 0.002 |
| Non-starchy Vegetable score | 0.74(0.54, 1.00) | 0.05 | 0.63(0.45, 0.87) | 0.01 | 0.69(0.49, 0.97) | 0.03 |
| Whole fruit score | 0.85(0.70, 1.03) | 0.10 | 0.67(0.55, 0.83) | <0.001 | 0.72(0.59, 0.88) | 0.002 |
| Fish and Shellfish score | 1.01(0.79, 1.28) | 0.96 | 1.00(0.78, 1.28) | 0.98 | 1.05(0.82, 1.35) | 0.69 |
| Nuts and Seeds score | 1.07(0.83, 1.38) | 0.59 | 0.87(0.67, 1.14) | 0.32 | 0.99(0.75, 1.31) | 0.95 |
| Added Fat, Unsaturated score | 0.72(0.43, 1.20) | 0.20 | 0.74(0.44, 1.25) | 0.26 | 0.79(0.47, 1.33) | 0.38 |
| Non-soy Legume score | 0.76(0.54, 1.07) | 0.11 | 0.79(0.55, 1.11) | 0.17 | 0.81(0.58, 1.12) | 0.20 |
| Soybean score | 0.80(0.43, 1.47) | 0.47 | 1.04(0.55, 1.95) | 0.91 | 1.28(0.70, 2.36) | 0.42 |
| Starchy Vegetable score | 0.77(0.59, 1.00) | 0.05 | 0.96(0.74, 1.24) | 0.73 | 1.00(0.77, 1.29) | 1.00 |
| Dairy score | 1.75(0.91, 3.35) | 0.09 | 2.44(1.27, 4.71) | 0.01 | 2.26(1.14, 4.49) | 0.02 |
| Red and Processed Meat score | 0.82(0.66, 1.01) | 0.07 | 0.93(0.73, 1.18) | 0.53 | 1.01(0.79, 1.28) | 0.96 |
| Poultry score | 1.26(0.98, 1.63) | 0.07 | 1.06(0.82, 1.37) | 0.66 | 1.04(0.80, 1.35) | 0.76 |
| Egg score | 0.87(0.62, 1.23) | 0.43 | 0.93(0.65, 1.32) | 0.68 | 0.99(0.69, 1.42) | 0.94 |
| Added Fat, Saturated score | 0.84(0.66, 1.07) | 0.15 | 1.03(0.80, 1.33) | 0.81 | 1.02(0.79, 1.31) | 0.90 |
| Added sugar score | 0.81(0.66, 0.99) | 0.04 | 0.69(0.56, 0.84) | <0.001 | 0.64(0.52, 0.78) | <0.001 |

Abbreviations: BMI, body mass index; CI, Confidence Interval; CVD, cardiovascular disease; DM, diabetes mellitus; OR, Odd Ratio; PHDI, Planetary Health Diet Index; PIR, poverty income ratio. The non-soy legume and soybean scores were entered as continuous variables per 5-point increase, while other PHDI components were entered per 10-point increase.

^a^. Crude model.

^b^. Adjusted for age, sex, race/ethnicity, marital status, PIR, education level.

^c^. Adjusted for age, sex, race/ethnicity, marital status, PIR, education level, BMI, physical activity, smoke, alcohol use, energy intake, hypertension, DM, and CVD.

**Supplementary Table 4. Associations between HDL-C and PHDI and self-reported kidney stones.**

|  | **X-M^a^** |  |  | **M-Y^b^** |  |
| --- | --- | --- | --- | --- | --- |
|  | ***β* (95%CI)^a^** | ***P* value** |  | **OR (95%CI)^c^** | ***P* value** |
| PHDI score | 0.88(0.67, 1.10) | <0.001 | HDL-C, mg/dL | 0.99(0.99, 1.00) | 0.02 |
| PHDI quintile |  |  | HDL-C quintile |  |  |
| Q1 | 0 [Reference] |  | Q1 | 1 [Reference] |  |
| Q2 | 0.98(0.11, 1.85) | 0.03 | Q2 | 1.17(0.98, 1.41) | 0.08 |
| Q3 | 2.32(1.52, 3.11) | <0.001 | Q3 | 1.08(0.87, 1.35) | 0.49 |
| Q4 | 2.11(1.09, 3.12) | <0.001 | Q4 | 0.91(0.70, 1.18) | 0.47 |
| Q5 | 3.34(2.41, 4.28) | <0.001 | Q5 | 0.90(0.69, 1.17) | 0.42 |

Note: X: exposure; M: mediating variable; Y: outcome. PHDI score was entered as a continuous variable per 10 points increase. The model was adjusted for age, sex, race/ethnicity, marital status, PIR, education level, BMI, physical activity, smoke, alcohol use, energy intake, hypertension, DM, and CVD. Abbreviations: BMI, body mass index; CI, Confidence Interval; CVD, cardiovascular disease; DM, diabetes mellitus; HDL-C, high-density lipoprotein cholesterol; OR, Odd Ratio; PHDI, Planetary Health Diet Index; PIR, poverty income ratio; Q, quintile.

^a^. X-M: Association between PHDI and HDL-C.

^b^. M-Y: Association between HDL-C and self-reported kidney stones.

**Supplementary Table 5. The mediating effect of HDL-C in the association between PHDI and self-reported kidney stones.**

|  | **Total effect(95CI%)** | ***P* value** | **Direct effect(95CI%)** | ***P* value** | **Indirect effect(95CI%)** | ***P* value** | **PM** | ***P* value** |
| --- | --- | --- | --- | --- | --- | --- | --- | --- |
| HDL-C | -0.010(-0.010, -0.010) | <0.001 | -0.009(-0.010, -0.009) | <0.001 | -0.0006(-0.0009, -0.000) | <0.001 | 6.0% | <0.001 |

Note: The direct effect (DE) signifies the influence of PHDI on self-reported kidney stones without mediation, while the indirect effect (IE) denotes the impact of PHDI on self-reported kidney stones through mediation. The proportion of mediation was computed by dividing IE by the total effect (TE).

Abbreviations: CI, Confidence Interval; HDL-C, high-density lipoprotein cholesterol; PHDI, Planetary Health Diet Index; PM, proportion mediation.

**Supplementary Table 6. Association between PHDI and self-reported kidney stones with multiple imputation analysis.**

|  | **Model 1^a^** |  | **Model 2^b^** |  | **Model 3^c^** |  |
| --- | --- | --- | --- | --- | --- | --- |
|  | **OR(95%CI)** | ***P* value** | **OR(95%CI)** | ***P* value** | **OR(95%CI)** | ***P* value** |
| **Before MI** |  |  |  |  |  |  |
| PHDI score^d^ | 0.94(0.89, 0.99) | 0.02 | 0.90(0.85, 0.95) | <0.001 | 0.92(0.87, 0.97) | 0.005 |
| Quintile |  |  |  |  |  |  |
| Q1 | 1[Reference] |  | 1[Reference] |  | 1[Reference] |  |
| Q2 | 1.10(0.87, 1.40) | 0.41 | 1.05(0.82, 1.34) | 0.7 | 1.08(0.84, 1.38) | 0.56 |
| Q3 | 0.86(0.69, 1.06) | 0.14 | 0.76(0.61, 0.95) | 0.02 | 0.78(0.63, 0.98) | 0.03 |
| Q4 | 0.82(0.67, 1.00) | 0.06 | 0.70(0.56, 0.87) | 0.001 | 0.74(0.60, 0.92) | 0.01 |
| Q5 | 0.80(0.64, 1.01) | 0.06 | 0.67(0.53, 0.86) | 0.002 | 0.75(0.58, 0.96) | 0.02 |
| **After MI** |  |  |  |  |  |  |
| PHDI score^d^ | 0.93(0.89, 0.98) | 0.01 | 0.89(0.84, 0.93) | <0.001 | 0.91(0.86, 0.96) | <0.001 |
| Quintile |  |  |  |  |  |  |
| Q1 | 1[Reference] |  | 1[Reference] |  | 1[Reference] |  |
| Q2 | 1.10(0.90, 1.36) | 0.34 | 1.03(0.83, 1.27) | 0.81 | 1.05(0.85, 1.30) | 0.65 |
| Q3 | 0.85(0.70, 1.03) | 0.10 | 0.74(0.60, 0.91) | 0.004 | 0.76(0.61, 0.94) | 0.01 |
| Q4 | 0.83(0.70, 1.00) | 0.05 | 0.69(0.57, 0.84) | <0.001 | 0.73(0.60, 0.89) | 0.001 |
| Q5 | 0.80(0.65, 0.98) | 0.03 | 0.65(0.52, 0.82) | <0.001 | 0.72(0.57, 0.92) | 0.01 |

Abbreviations: BMI, body mass index; CI, Confidence Interval; CVD, cardiovascular disease; DM, diabetes mellitus; OR, Odd Ratio; PHDI, Planetary Health Diet Index; PIR, poverty income ratio; Q, quintile; MI, multiple imputation.

^a^. Crude model.

^b^. Adjusted for age, sex, race/ethnicity, marital status, PIR, education level.

^c^. Adjusted for age, sex, race/ethnicity, marital status, PIR, education level, BMI, physical activity, smoke, alcohol use, energy intake, hypertension, DM, and CVD.

^d^. PHDI score was entered as a continuous variable per 10 points increase.
